# Hemopericardium in a Sirolimus-Treated Renal Transplant Patient

**DOI:** 10.7759/cureus.9508

**Published:** 2020-08-01

**Authors:** Kevin Groudan, Khalid Sawalha, Anusha G Bhat, Ahmed Eltanbedawi, Paurush Ambesh

**Affiliations:** 1 Internal Medicine, Baystate Medical Center, Springfield, USA; 2 Internal Medicine, University of Massachusetts Medical School Baystate, Massachusetts, USA; 3 Cardiovascular Medicine, University of Massachusetts Medical School Baystate, Massachusetts, USA

**Keywords:** hemopericardium, sirolimus, renal transplant, immunosuppression

## Abstract

Sirolimus is an immunosuppressant frequently prescribed to prevent graft-vs-host disease in renal transplant patients. Pericardial effusion is recognized as a rare and potentially lethal side effect of this medication. Hemopericardium, specifically, is an even rarer complication that has yet to be reported in the literature. We report the first case of sirolimus-induced hemopericardium in a renal transplant patient.

## Introduction

Sirolimus, sold under the brand name Rapamune, is an immunosuppressive drug that prevents graft-vs-host disease in renal transplant recipients [1]. Pericardial effusion is a rare but potentially fatal side effect of this medication [2]. Hemoperricardium, specifically, has yet to be reported in a sirolimus-treated renal transplant patient. We report a 62-year-old renal transplant recipient, on sirolimus for nine years without major complications, who presented to our hospital with chest pain and was found to have a large hemopericardium.

## Case presentation

A 62-year-old man with a history of end-stage renal disease, status post renal transplant in 2009, on prednisone and sirolimus for immunosuppression, Stage 4 chronic kidney disease (CKD) of his transplant kidney, and New York Heart Association (NYHA) class III congestive heart failure (CHF), presented to our hospital with acute onset chest pain and shortness of breath. He was afebrile and hemodynamically stable. On physical exam, he had clear breath sounds, no leg swelling or jugular venous distention, and no murmurs, rubs or gallops. His complete blood count was unremarkable, electrolytes were stable, creatinine was baseline at 2.7 mg/dL, thyroid-stimulating hormone (TSH) was normal, and anti-nuclear antibody screen, rheumatoid factor, and cytoplasmic and perinuclear anti-neutrophil cytoplasmic antibodies (ANCAs) were negative.

Given concern for acute coronary syndrome, an electrocardiogram was obtained which demonstrated questionable ST elevations in the inferior leads. Troponin was elevated at .03 ng/mL, possibly from CKD. Given concern for pneumonia versus CHF exacerbation, chest x-ray was obtained which demonstrated a large cardiac silhouette. Given these findings, a bedside cardiac ultrasound was performed which demonstrated a very large pericardial effusion. Interventional radiology was consulted and took the patient for an emergent pericardial effusion drainage. The patient remained hemodynamically stable without cardiac tamponade physiology.

Successful CT-guided tube pericardiostomy was performed with removal of 1,900cc of extremely hemorrhagic fluid. The pericardial drainage catheter was sutured in place to allow for further drainage. The pericardial fluid collection was sent for cytology and culture. The fluid appearance was red and contained over 50,000 red blood cells (RBCs) per cubic millimeter; however, white blood cells were within normal limits. Cytology revealed primarily blood and no malignant cells. Culture did not grow organisms.

**Figure 1:**
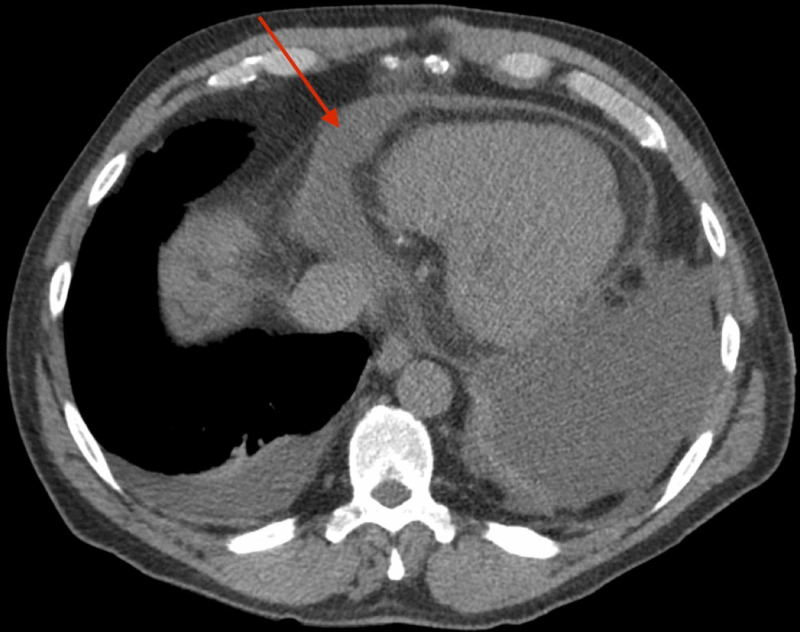
CT chest with contrast confirmed an interval decrease in the size of the pericardial effusion following catheter drainage

Sirolimus was held and nephrology consulted for concern for sirolimus-associated pericardial effusion. Trough sirolimus level returned high at 27.6 ng/mL (normal is 3.0-20.0). Nephrology switched the patient’s sirolimus, which he had tolerated for nine years, to tacrolimus and mycophenolate mofetil (MMF). After the pericardial drain was removed, an echocardiogram was performed, revealing residual moderate pericardial effusion near the right atrial free wall, no evidence of cardiac tamponade, and moderate to severely reduced left and right ventricular systolic functions. CT chest with contrast confirmed an interval decrease in the size of the pericardial effusion following catheter drainage (Figure 1). The patient was discharged home with sirolimus discontinued and tacrolimus and MMF ordered in its place.

## Discussion

Sirolimus binds to FK-binding protein 12 to inhibit mammalian target of rapamycin (mTOR), a serine/threonine protein kinase that regulates growth, proliferation, and cellular metabolism [1]. Initially isolated as an anti-fungal agent, sirolimus was later discovered to have potent immunosuppressive properties [2]. It is most commonly used to prevent acute rejection in transplant recipients but also can treat tuberous sclerosis, psoriasis, and malignancy because of its anti-proliferative properties. Clinicians should be aware of its adverse reactions prior to prescribing it. Common side effects include diarrhea, nausea, constipation, skin rash, and headache. Rare but life-threatening side effects include lymphoma and other malignancy, interstitial pneumonia, lymphocytic meningitis, and pericardial effusion [3].

The differential for pericardial effusion is broad, including endocrine, immunologic, infectious, neoplastic, drug-related and idiopathic causes. Our patient denied any flu-like symptoms or a recent history of viral infection. Additionally, his pericardial fluid culture returned negative. A normal TSH ruled out hypothyroidism. His rheumatologic workup was negative. The cytology report did not reveal malignant cells, ruling out neoplasm. Uremia did not seem likely because his blood urea nitrogen was not acutely elevated, and he lacked uremic symptoms, such as fatigue, peripheral neuropathy, or pruritis. Tuberculosis (TB) was considered, given his drainage of hemorrhagic fluid and transplant recipients have a higher risk of Mycobacterium tuberculosis infection than the general population. However, his fluid culture was negative, and he lacked other risk factors for acquiring TB. Given his negative work-up and the known association between sirolimus and pericardial effusion in post-transplant patients, an adverse drug reaction was most probable.

Literature on pericardial effusion in sirolimus-treated renal transplant patients remains limited. While sirolimus-treated cardiac transplant patients have a reported incidence of pericardial effusion of 9%-30%, the largest single center report of pericardial effusion in renal transplant patients noted only 19 of 792 cases studied were affected [4,5]. In these patients, the mean duration of sirolimus treatment prior to symptoms was quite long at 5.06 years (ranging from 0.5-9.8), consistent with our patient’s late presentation, approximately nine years after starting sirolimus. Also of note, our patient’s sirolimus level was markedly higher than the patients in that study; 27.6 ng/mL at the time of his diagnosis as compared to 5.19-7.47 in that study. It is unclear if this has any relationship to the severity of his presentation.

The mechanism leading to sirolimus-associated pericardial effusion is still unclear. Lymphedema, an increasingly reported side effect of sirolimus, has been suggested as a predisposing factor for the development of pericardial effusion [6]. There are numerous case reports of lymphedema associated with sirolimus use [7-10]. De Bartolomeis et al. specifically reported a renal transplant patient who had lymphedema, ascites and pleural effusions associated with his pericardial effusion [11]. The pathophysiology of this phenomenon is not well understood. Nevertheless, lymphedema was not found in our patient, and his pericardial effusion drained grossly hemorrhagic fluid. Studies have shown that suppression of mTOR by sirolimus can induce nuclear factor (NF)-kB, a regulator of inflammation [12]. This pro-inflammatory effect could explain the development of pericardial effusion secondary to serositis.

## Conclusions

In conclusion, while sirolimus-associated pericardial effusion has been reported in the literature, we present the first case of hemopericardium in a renal transplant patient. High suspicion and detailed workup are needed so that clinicians do not miss this rare complication.
